# *Clinopodium nepeta* Essential Oil and Its Major Constituents Pulegone and Menthone: Composition and Activity Against *Verticillium dahliae*, *Phytophthora infestans*, and *Macrophomina phaseolina*

**DOI:** 10.1002/cbdv.71781

**Published:** 2026-09-30

**Authors:** Mohamed Taibi, Amine Elbouzidi, Mounir Haddou, Abdessamad Beraich, Abdellah Baraich, Reda Bellaouchi, Mohamed Addi, Khalid Chaabane, Bassem Jaouadi, Nadia Zarai, Bouchra El Guerrouj, Abdeslam Asehraou

**Affiliations:** ^1^ Laboratory of Bioresources, Biotechnology, and Sustainable Development (L2BD), Faculty of Sciences Mohammed Premier University Oujda Morocco; ^2^ Laboratoire d'Amélioration des Productions Agricoles, Biotechnologie et Ecophysiologie (LAPABE), Faculté des Sciences Université Mohammed Premier Oujda Morocco; ^3^ Physical Chemistry of Natural Resources and Process Team, Laboratory of Applied Chemistry and Environment (LCAE) Faculty of Sciences, Mohammed First University Oujda Morocco; ^4^ Laboratoire des Biotechnologies Microbiennes et Enzymatiques et Biomolécules (LBMEB) Centre de Biotechnologie de Sfax (CBS), Université de Sfax (USF) Sfax Tunisia

**Keywords:** antifungal activity, biopesticides, *Clinopodium nepeta*, essential oils, menthone, pulegone, terpenoids

## Abstract

Fungal and oomycete phytopathogens account for 10%–20% of annual global crop losses, and the escalating resistance to synthetic fungicides has intensified demand for plant‐derived alternatives. The essential oil (EO) of *Clinopodium nepeta* (L.) Kuntze, obtained by hydrodistillation from aerial parts harvested in northeastern Morocco, was profiled by gas chromatography–mass spectrometry (GC–MS) and evaluated for antifungal and anti‐oomycete activity against *Verticillium dahliae*, *Phytophthora infestans*, and *Macrophomina phaseolina* using broth microdilution minimum inhibitory concentration (MIC) assays. The EO was dominated by two oxygenated monoterpene ketones, pulegone (42.16%) and menthone (41.43%), which were additionally evaluated as isolated compounds. The whole EO yielded MIC values of 31.25 µg/mL (*P. infestans*), 62.5 µg/mL (*V. dahliae*), and 125 µg/mL (*M. phaseolina*). Both isolated constituents outperformed the native matrix by 2‐ to 16‐fold, revealing antagonistic interactions within the complex EO. Pulegone showed superior activity against *V. dahliae* and *P. infestans*, while menthone was more potent against *M. phaseolina*, a selectivity pattern consistent with the greater electrophilic reactivity of pulegone's α,β‐unsaturated ketone (Michael acceptor) moiety. These findings identify pulegone and menthone as priority biopesticide candidates, pending formulation optimization, in planta validation, and toxicological assessment of pulegone under field‐exposure conditions. Because the GC–MS analysis was performed on an achiral stationary phase, the enantiomeric composition of the oil was not determined; its constituents are therefore reported without stereodescriptors, and the compounds assayed as isolated agents were the commercial reference standards (R)‐(+)‐pulegone and (2S,5R)‐(−)‐menthone. The structure–activity relationship described here is consequently constitutional rather than configurational.

## Graphical abstract

Chemical fractionation of *Clinopodium nepeta* essential oil reveals pulegone and menthone as markedly more potent antifungal isolates than the whole oil, with activity varying among pathogens. Pulegone exhibits superior efficacy against *Verticillium dahliae* and *Phytophthora infestans*, whereas menthone shows enhanced activity against *Macrophomina phaseolina*, highlighting pathogen‐specific selectivity and complementary mechanisms.

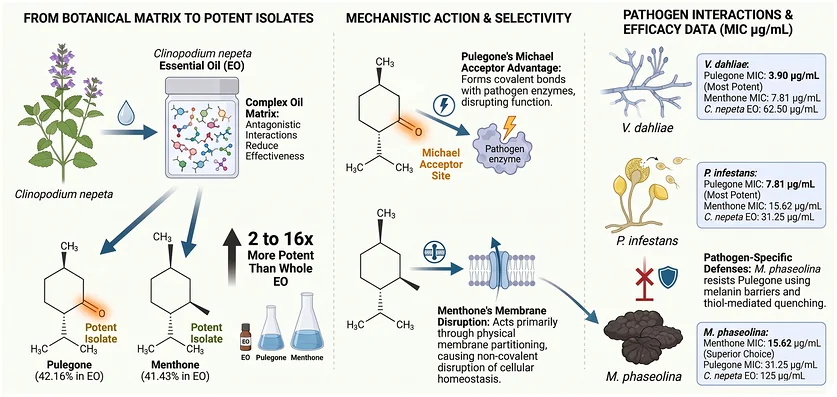

## 1 Introduction

Agricultural production worldwide is threatened by a diverse community of plant‐pathogenic microorganisms. Fungal and oomycete pathogens collectively account for an estimated 10%–20% reduction in global crop yields annually, with losses in staple crops such as potato, tomato, cotton, and legumes running to tens of billions of euros per year [1, 2]. Despite decades of chemical control efforts, these pathogens continue to evade sustainable management, underscoring the urgent need for innovative, mode‐of‐action‐diversified crop protection strategies [3]. Among the most economically destructive phytopathogens are the soilborne agents *Verticillium dahliae*, *Phytophthora infestans*, and *Macrophomina phaseolina*, each presenting distinct biological and control challenges.

*Verticillium dahliae* Kleb. is an asexual ascomycete responsible for Verticillium wilt, one of the most destructive soilborne vascular diseases globally. Its host range encompasses more than 400 dicotyledonous plant species, and economic losses in cotton alone are estimated at 10%–35% of yield per season [2, 4]. The pathogen colonizes host xylem vessels, physically blocking water transport and triggering defensive occlusions, and produces melanized microsclerotia that can persist in soil for decades, severely limiting sanitation strategies [5]. Notably, no single fungicide class has proven broadly effective against *V. dahliae* across its diverse host range, and resistance to benzimidazoles and demethylation inhibitors has been increasingly documented in field populations [6].

*Phytophthora infestans* (Mont.) de Bary is the causative agent of late blight of potato and tomato, historically responsible for the Irish famine and currently causing annual economic losses valued at several billion euros worldwide [7]. *Phytophthora infestans* is not a true fungus but an oomycete (class Oomycota, kingdom Stramenopiles), a distinction with profound mechanistic implications [8]. Unlike true fungi, oomycetes synthesize cellulose rather than chitin as the primary cell wall polysaccharide, and their plasma membranes are enriched in fucosterol rather than ergosterol [9]. Conventional fungicides targeting ergosterol biosynthesis (azoles, polyenes) or chitin synthase (benzimidazoles) therefore show inherently reduced or absent efficacy against oomycetes, necessitating dedicated oomyceticides such as metalaxyl or mefenoxam [10, 11].

*Macrophomina phaseolina* (Tassi) Goid. is a polyphagous necrotrophic soilborne fungus infecting over 500 plant species and causing charcoal rot, a disease of exceptional severity under conditions of heat stress and drought that simultaneously weaken host defenses and promote pathogen growth [12]. The pathogen produces heavily melanized microsclerotia that provide extraordinary physical and chemical resistance to environmental stressors and antifungal agents [13].

Current disease management relies predominantly on synthetic fungicides and oomyceticides. Despite their near‐term efficacy, repeated applications have rapidly driven resistance emergence [14, 15]. Metalaxyl resistance in *P. infestans* was documented within a decade of the compound's commercial introduction, and the regulatory landscape is tightening significantly [16]. Under EU Regulation (EC) No 1107/2009, more than 70 active substances were withdrawn from the European market between 2007 and 2017, and Morocco's export‐oriented agricultural sector faces analogous pressures as trading partners impose stricter maximum residue level (MRL) requirements [17, 18, 19]. These converging drivers have reinvigorated interest in plant‐derived, biologically based crop protection alternatives. In parallel with regulatory restrictions on conventional pesticides, global agriculture is increasingly adopting integrated pest management (IPM) strategies that prioritize environmentally compatible disease‐control solutions. Plant‐derived bioactive compounds are receiving particular attention because of their rapid biodegradability, reduced environmental persistence, and generally lower ecotoxicological impact compared with many synthetic fungicides. Consequently, the identification of natural compounds with broad‐spectrum efficacy against taxonomically distinct phytopathogens has become a major research priority within sustainable crop‐protection programs [20, 21].

Plant essential oils (EOs) represent structurally diverse mixtures of low‐molecular‐weight volatile compounds, principally terpenes, terpenoids, and phenylpropanoids, produced by aromatic plants as constitutive and inducible chemical defenses [22]. Their biological activity typically arises from multiple mechanisms operating simultaneously on membrane integrity, enzyme function, and metabolic processes, which intrinsically limits the probability of target‐specific resistance development compared with single‐mechanism synthetic fungicides [23, 24]. The family Lamiaceae is particularly rich in EO‐producing genera with documented antifungal activity, including *Thymus*, *Origanum*, *Mentha*, *Satureja*, and *Clinopodium*, genera whose EOs are enriched in oxygenated monoterpenes that disrupt microbial membrane homeostasis [25, 26, 27].

*Clinopodium nepeta* (L.) Kuntze (syn. *Calamintha nepeta*; lesser calamint) is a perennial aromatic herb native to the Mediterranean basin and widely distributed across North Africa, including Morocco. Its EO chemistry is markedly influenced by geographic origin, phenological stage, and altitude [28, 29]. Populations from Tunisia and Turkey have been characterized by piperitone oxide and piperitenone oxide as dominant constituents, whereas the Moroccan population investigated in this study belongs to the pulegone/menthone chemotype, a chemical variant with distinctive pharmacological properties [30, 31]. This chemotype has recently been confirmed in a parallel study of *C. nepeta* EO from the same region (pulegone 42.3%).

Pulegone (p‐menth‐4(8)‐en‐3‐one) and menthone (p‐menthan‐3‐one) are oxygenated monoterpene ketones biosynthetically related through the pulegone reductase (PulR) pathway [32, 33]. Both have been reported to exhibit antimicrobial, antifungal, insecticidal, and anti‐inflammatory properties in various biological systems [34, 35]. Of particular mechanistic significance, pulegone carries an α,β‐unsaturated cyclic ketone (enone) system, a Michael acceptor site that confers intrinsic electrophilicity absent in the fully saturated ketone of menthone [36, 37]. This structural difference is central to interpreting the differential bioactivity observed across pathogens in the present study. Throughout this article the unprefixed names pulegone and menthone denote the constituents of the EO, whose absolute configuration was not established; stereodescriptors are used only for the defined reference compounds employed in the bioassays (Section 2.3).

Despite growing literature on *C. nepeta* EO bioactivity, a critical gap persists: most studies assess the whole EO as a composite mixture without evaluating the individual contributions of its dominant constituents. This limitation is not trivial: The activity of a whole EO does not necessarily reflect the sum of its component activities, because antagonistic, synergistic, or matrix effects within the mixture can substantially modify, and in many cases reduce the potency of individual compounds [38, 39]. Surprisingly, only a handful of studies on Lamiaceae EOs have systematically compared whole EOs with their purified major constituents under identical bioassay conditions, and none—to our knowledge—has done so for *C. nepeta* against a panel including both true fungi and an oomycete. Constituent deconvolution, testing isolated major compounds alongside the whole EO under identical conditions, is essential to understand the mechanistic basis of activity and to rationally design enriched biopesticide formulations. Furthermore, to our knowledge, the specific combination of *V. dahliae*, *P. infestans*, and *M. phaseolina* as target organisms has not been employed in any prior study of *C. nepeta* EO. The inclusion of *P. infestans* as an oomycete adds a mechanistically distinctive dimension that most purely mycocidal studies overlook. The chemical composition reported below is, we readily acknowledge, confirmatory: the pulegone/menthone chemotype of *C. nepeta* is already documented. What has not previously been reported is the direct comparison of the whole oil with its isolated major constituents under identical assay conditions, and the resulting observation that the constituents are the more potent agents, a result that is opposite in direction to the synergy usually assumed for essential‐oil matrices, and that carries a concrete formulation consequence.

Although several studies have reported the antimicrobial properties of *C. nepeta* EOs, very few investigations have attempted to dissect the contribution of individual constituents to the overall biological activity. Furthermore, comparative studies simultaneously targeting true fungi and oomycetes remain extremely limited. The present work addresses these gaps by integrating phytochemical characterization with constituent‐specific bioactivity assessment, thereby providing a mechanistic framework for understanding how chemical composition influences pathogen selectivity and inhibitory potency. The objectives of this work were to characterize, by GC–MS with retention index confirmation, the chemical composition of *C. nepeta* EO from northeastern Morocco, to evaluate its in vitro antifungal and anti‐oomycete activity against *V. dahliae*, *P. infestans*, and *M. phaseolina* in broth microdilution MIC assays, and to directly compare the activity of the whole EO with that of its two major isolated constituents, pulegone and menthone, in order to determine whether constituent interactions are antagonistic or synergistic and to identify the molecular drivers of the observed selectivity patterns.

## 2 Materials and Methods

### 2.1 Plant Material and Essential Oil Extraction

Aerial parts of *C. nepeta* (L.) Kuntze were supplied by a registered herbal materials vendor (STE ANGAD NATURE, Oujda, Morocco; Common Business Identifier 000031254000042), which sourced the plant material from local cultivation. Whole plant specimens including leaves, stems, and flowers were retained for taxonomic authentication, which was performed by Prof. Mohammed Addi (botanist, Faculty of Sciences, Mohammed First University, Oujda) on the basis of morphological characters. A voucher specimen bearing the registration number CN‐2024‐015 was deposited in the Herbarium of the Faculty of Sciences, Mohammed First University, Oujda, Morocco. The same authenticated batch was used in our previously published studies on this EO [31]. The plant material was air‐dried at ambient temperature in the shade for 14 days prior to extraction. EO was obtained by hydrodistillation of dried aerial parts (100 g) using a modified Clevenger‐type apparatus for 3 h. The oil was separated from the aqueous condensate, dried over anhydrous sodium sulfate, and stored at 4°C in amber vials under nitrogen atmosphere until analysis. Extraction yield was 3.72% (*w/w*). Botanical identification was performed on entire flowering specimens rather than on processed or fragmented commercial material; the vendor supplied bulk plant material only and played no part in the taxonomic determination. The voucher specimen CN‐2024‐015 is permanently deposited and is available on request, so that the identity of the material can be independently re‐examined and the experiments reproduced.

### 2.2 GC–MS Analysis

Chemical profiling was performed using a Shimadzu QP2010 gas chromatograph–mass spectrometer (Shimadzu Corporation, Kyoto, Japan), equipped with a Restek Rtx‐5MS capillary column (30 m × 0.25 mm i.d. × 0.32 µm film thickness; 5% diphenyl/95% dimethylpolysiloxane stationary phase). Helium (≥99.999% purity) served as carrier gas at a constant column flow of 1.0 mL/min. The oven temperature program was: 50°C (held 1 min), ramp at 10°C/min to 250°C. Mass spectra were acquired in full‐scan mode over 40–300 m/z at 70 eV electron ionization. Samples (diluted 1:20 v/v in anhydrous ethanol, 1 µL injection volume) were introduced in split mode (split ratio 1:20). All analyses were performed in triplicate. The Rtx‐5MS phase (5% diphenyl/95% dimethylpolysiloxane) is achiral and does not resolve enantiomers; no enantioselective (cyclodextrin‐based) column was available for this study. Enantiomeric ratios were therefore not measured, and the constituents listed in Table 1 are reported without stereodescriptors.

**TABLE 1:** Chemical composition of *C. nepeta* essential oil obtained by hydrodistillation.

Peak no.	Compound	Compound class	LRI calculated	LRI literature	Method	Relative area (%)
1	α‐Pinene	Monoterpene hydrocarbon	939	939	MS/RI	0.38
2	β‐Pinene	Monoterpene hydrocarbon	979	979	MS/RI	0.38
3	β‐Myrcene	Monoterpene hydrocarbon	991	991	MS/RI	0.16
4	3‐Octanol	Aliphatic alcohol	994	994	MS/RI	0.07
5	Limonene	Monoterpene hydrocarbon	1029	1029	MS/RI	3.30
6	β‐Phellandrene	Monoterpene hydrocarbon	1030	1030	MS/RI	0.14
7	4‐Isopropyl‐1,3‐cyclohexanedione	Oxygenated monoterpene	1139	1140	MS/RI	0.54
8	Menthone	Oxygenated monoterpene	1152	1152	MS/RI/Std	41.43
9	Unidentified	—	1163	—	—	0.91
10	Neoisomenthol	Oxygenated monoterpene	1166	1163	MS/RI	9.25
11	Unidentified	—	1205	—	—	0.17
12	Pulegone	Oxygenated monoterpene	1237	1237	MS/RI/Std	42.16
13	Thymol	Monoterpenoid phenol	1290	1290	MS/RI	0.71
14	Caryophyllene oxide	Oxygenated sesquiterpene	1581	1582	MS/RI	0.40
	**Oxygenated monoterpenes**					**93.38**
	**Monoterpene hydrocarbons**					**4.36**
	**Monoterpenoid phenols**					**0.71**
	**Oxygenated sesquiterpenes**					**0.40**
	**Aliphatic alcohols**					**0.07**
	**Total identified**					**98.92**
	**Unidentified**					**1.08**
	**Total**					**100.00**

Compounds are listed in order of elution, that is by increasing calculated linear retention index (LRI).

Identification method: MS/RI = mass‐spectral library match corroborated by agreement of the calculated LRI with the literature value; MS/RI/Std = additionally confirmed by co‐injection of an authentic standard. Peaks for which a mass‐spectral match alone was available and could not be corroborated are reported as unidentified. Constituents are named without stereodescriptors because the separation was performed on an achiral stationary phase (Section 2.2). Relative areas are uncorrected total‐ion‐chromatogram peak areas.

Compound identification was based on two complementary criteria: comparison of experimental mass spectra with those in the National Institute of Standards and Technology (NIST)/Wiley spectral library, retaining only matches with similarity indices ≥95%, and comparison of calculated linear retention indices (LRIs), obtained using a homologous n‐alkane series (C8–C24) under identical chromatographic conditions, with literature LRI values from the Adams essential‐oil database. Relative peak area percentages were calculated from total‐ion chromatograms without detector‐response correction. Because a mass‐spectral library match is not by itself a sufficient criterion of identity, assignments supported by library matching but corroborated neither by an LRI within ±5 units of the literature value nor by an authentic standard are reported as unidentified. On re‐evaluation of the raw spectra against this criterion, the two minor peaks previously assigned as 1,3,4‐trimethyl‐3‐cyclohexene‐1‐carboxaldehyde and 4,4,7‐trimethyloct‐5‐enal were judged unreliable, since neither compound is documented in *Clinopodium* or *Calamintha* EOs and no authentic standard was available for co‐injection; both are now listed as unidentified. Authentic standards of pulegone and menthone were available in this laboratory (Section 2.3) and the identity of the two dominant peaks was confirmed by co‐injection under identical chromatographic conditions.

### 2.3 Chemicals

(R)‐(+)‐Pulegone [(5R)‐5‐methyl‐2‐(propan‐2‐ylidene)cyclohexan‐1‐one; CAS 89‐82‐7, ≥97%] and (2S,5R)‐(−)‐menthone [(2S,5R)‐5‐methyl‐2‐(propan‐2‐yl)cyclohexan‐1‐one, equivalent to (1R,4S)‐p‐menthan‐3‐one; CAS 14073‐97‐3, ≥99%] were obtained from Sigma‐Aldrich, St. Louis, MO, USA. Stock solutions of each compound were prepared in dimethyl sulfoxide (DMSO) with the final solvent concentration in assay wells 2.5% v/v DMSO, a concentration independently verified to produce no growth inhibition (solvent control). All reagents were of analytical grade.

### 2.4 Microbial Strains

Three phytopathogenic microorganisms were used: *V. dahliae* Kleb., *P. infestans* (Mont.) de Bary, and *M. phaseolina* (Tassi) Goid. Note: *P. infestans* is an oomycete (Stramenopiles), not a true fungus; it is included here because it is a primary field‐relevant pathogen against which biopesticide candidates must be evaluated, and the in vitro broth microdilution MIC format is applicable across kingdoms. Mechanistic interpretations, however, must account for its distinct cell wall (cellulose, not chitin) and membrane sterol composition (fucosterol, not ergosterol).

### 2.5 Minimum Inhibitory Concentration (MIC) Assay

Antifungal and anti‐oomycete activity was assessed by broth microdilution in 96‐well flat‐bottomed microtiter plates adapted from Clinical and Laboratory Standards Institute (CLSI) document M38‐A2 for filamentous fungi. Growth medium: Potato Dextrose Broth (PDB) for true fungi, V8 broth for *P. infestans*. Stock solutions of the EO, pulegone, and menthone, were prepared at 10 mg/mL in DMSO and serially diluted two‐fold across the concentration range of 3.90–250 µg/mL in a total well volume of 200 µL. Each well was inoculated with a standardized suspension of 1 × 10^4^ conidia/mL for *V. dahliae* and *M. phaseolina*; 1 × 10^3^ sporangia/mL for *P. infestans*, prepared in each medium. Plates were sealed and incubated at 25°C for *V. dahliae* and *M. phaseolina*; 20°C for *P. infestans* for 72 h for true fungi, 96 h for *P. infestans*, without shaking. Negative controls contained medium plus inoculum (growth control); solvent controls contained the maximum vehicle concentration used. MIC was defined as the lowest compound concentration at which no visible turbidity or mycelial growth was observed by unaided visual inspection, confirmed by plate reader absorbance at 595 nm where applicable. To ensure reproducibility and minimize experimental bias, all treatments and controls were randomly distributed across microplate wells. Growth inhibition was independently assessed by two investigators, and discrepancies were resolved by consensus. These procedures were implemented to enhance the robustness and reliability of MIC determination. All assays were conducted in three independent experiments. MIC values are reported as geometric means ± SD across biological replicates.

### 2.6 Statistical Analysis

All statistical analyses were performed using GraphPad Prism version 10.0 (GraphPad Software, San Diego, CA, USA). Data are expressed as mean ± standard deviation (SD) of three independent replicates (*n* = 3). Differences in MIC values among test agents were assessed by one‐way analysis of variance (ANOVA) followed by Tukey's honestly significant difference (HSD) post‐hoc test for pairwise comparisons. MIC values were log_2_‐transformed prior to analysis to satisfy normality requirements inherent to geometric dilution series. Statistical significance was set at *p* < 0.05.

## 3 Results and Discussion

### 3.1 Chemical Composition and Chemotype Identity

GC–MS analysis of the *C. nepeta* EO resolved 14 peaks, of which 12 were identified, together accounting for 98.92% of the total composition (Table 1 and Figure S1). The EO is characterized by a marked predominance of oxygenated monoterpenes, which collectively constitute 93.38% of the total composition, a profile characteristic of the pulegone/menthone chemotype of this species. Pulegone (42.16%) and menthone (41.43%) co‐dominate the profile, together accounting for approximately 83.6% of the total oil. Their near‐equimolar ratio reflects balanced flux through the pulegone reductase pathway at the phenological stage of harvest and distinguishes this Moroccan population sharply from the piperitone oxide/piperitenone oxide‐dominant chemotypes reported in Tunisia (piperitone oxide 51.7%, piperitenone oxide 23.4%) and Turkey [30]. The chemical profile reported here closely parallels that recently published by Taibi et al. (2025) on *C. nepeta* from the same geographic region of northeastern Morocco (pulegone as dominant compound at 42.3%), confirming the chemotype stability within this population across independent harvests [2]. To our knowledge, this is the first report of near‐equimolar pulegone and menthone in *C. nepeta* from northeastern Morocco, reinforcing the existence of a stable chemotype distinct from Mediterranean piperitone‐oxide‐dominant populations. Neoisomenthol (9.25%) constitutes the most significant minor component, followed by limonene (3.30%). Additional oxygenated constituents included thymol (0.71%), 4‐isopropyl‐1,3‐cyclohexanedione (0.54%), and caryophyllene oxide (0.40%). Monoterpene hydrocarbons were detected only in minor proportions (α‐pinene 0.38%, β‐pinene 0.38%, β‐myrcene 0.16%, and β‐phellandrene 0.14%), and the aliphatic alcohol 3‐octanol was present at trace level (0.07%). Two minor peaks previously reported as 1,3,4‐trimethyl‐3‐cyclohexene‐1‐carboxaldehyde (0.91%) and 4,4,7‐trimethyloct‐5‐enal (0.17%) rested on library matching alone. Neither compound is documented in *Clinopodium* or *Calamintha* EOs, and in the absence of authentic standards these assignments could not be defended; both peaks are accordingly reported as unidentified (Table 1, peaks 9 and 11) and together represent 1.08% of the total oil. Their reassignment does not affect any conclusion of this work, since neither peak entered the bioactivity interpretation. For the same reason of analytical rigor, and because the separation was achiral, the earlier designation D‐limonene has been withdrawn in favor of limonene.

The predominance of oxygenated monoterpenes (93.38%) is noteworthy because compounds belonging to this chemical class are generally recognized as exhibiting stronger antimicrobial activities than monoterpene hydrocarbons owing to their increased polarity and capacity to interact with biological membranes and intracellular targets [40]. Consequently, the chemical profile observed in the present study may partly explain the remarkable anti‐phytopathogenic activity of the investigated oil.

The *C. nepeta* population from northeastern Morocco belongs unambiguously to the pulegone/menthone chemotype, with these two compounds alone accounting for approximately 83.6% of the oil. Intraspecific chemical variability in *C. nepeta* is well‐documented and reflects a combination of genetic population structure and phenotypic plasticity driven by local climate, soil mineralogy, altitude, and phenological stage [41]. The pulegone/menthone chemotype, characterized by the sequential‐biosynthesis pair pulegone → menthone via pulegone reductase, has been documented in related Lamiaceae genera including *Schizonepeta* spp. (where this chemotype drives anti‐inflammatory and antifungal activity) and *Mentha pulegium* (pennyroyal, whose EO is characteristically rich in pulegone and isomenthone) [42, 43]. The near‐equimolar abundance of both precursor and product in the present sample suggests balanced pulegone reductase activity and may be sensitive to the specific developmental stage at which aerial parts were harvested [41]. A comparison with published *C. nepeta* data from the same region is particularly instructive: Taibi et al. recently reported a pulegone content of 42.3% in *C. nepeta* EO from northeastern Morocco, virtually identical to the 42.16% recorded here [31]. This cross‐study consistency confirms that the pulegone/menthone chemotype is a stable and reproducible feature of this geographic population, not an artifact of extraction or seasonal variation, and provides a solid basis for comparative bioactivity analysis. The 42.3% figure quoted for comparison is that reported by Taibi et al. for *C. nepeta* EO from the same region (*Skin Research and Technology* 31, e70191; https://doi.org/10.1111/srt.70191), in which 13 constituents and 91.9% oxygenated monoterpenes were recorded.

A limitation of the present chemical characterization must be stated explicitly. The stationary phase used here is achiral, so the constituents of the oil were identified at the level of constitution only, and their enantiomeric composition was not determined [41]. The distinction is not academic. Enantiomeric ratios in Lamiaceae oils are neither uniform nor invariably unity: enantioselective and multidimensional gas chromatography of mint oils shows pulegone present with a strong but incomplete excess of one enantiomer, menthone and isomenthone recovered with very high but separately determined enantiomeric purity, and limonene present predominantly as the (S)‐(−) rather than the (R)‐(+) antipode [42, 43]. The routine labeling of the limonene peak of a Lamiaceae oil as D‐limonene on the basis of an achiral separation is, on that evidence, unsafe. Because enantiomeric distribution is set by the stereoselectivity of the biosynthetic enzymes, pulegone reductase reduces the two pulegone enantiomers at different rates and partitions them differently between menthone and isomenthone, the enantiomeric composition of *C. nepeta* cannot in any case be inferred from that of *Mentha* [41]. We therefore make no claim regarding the configuration of the oil constituents. The compounds assayed as isolated agents were the commercial reference standards (R)‐(+)‐pulegone and (2S,5R)‐(−)‐menthone, whose configuration is certified by the supplier, and it is to these defined stereoisomers alone that the MIC values in Table 2 refer. Determining whether the activity of the oil is attributable to these particular enantiomers requires enantioselective GC on a cyclodextrin stationary phase together with bioassay of both antipodes; we identify this as the necessary next step and have not undertaken it here [31].

**TABLE 2:** Antifungal and anti‐oomycete activity, minimum inhibitory concentrations (µg/mL) of *C. nepeta* EO, pulegone, menthone, and bifonazole (antifungal standard) against the three target pathogens.

Test agent	*V. dahliae* (ascomycete)	*P. infestans* (oomycete)	*M. phaseolina* (ascomycete)
*C. nepeta* EO	62.50 ± 1.00	31.25 ± 0.25	125 ± 0.25
Pulegone	3.90 ± 0.25	7.81 ± 0.25	31.25 ± 0.00
Menthone	7.81 ± 0.10	15.62 ± 0.25	15.62 ± 1.00
Bifonazole (antifungal standard)	250.00 ± 0.00	500 ± 0.00	250 ± 0.00

Bold values indicate the lowest MIC recorded within each column. Compounds are named without stereodescriptors; the agents assayed were the commercial standards (R)‐(+)‐pulegone and (2S,5R)‐(−)‐menthone (Section 2.3).

Values are means ± SD of *n* = 3 independent experiments. EO = essential oil.

*Note: P. infestans* is an oomycete, not a true fungus; MIC values reflect anti‐oomycete rather than antifungal activity for this organism.

### 3.2 Antifungal and Anti‐Oomycete Activity

The inhibitory activity of the *C. nepeta* EO, pulegone, menthone, and bifonazole (antifungal standard) against all three target pathogens is summarized in Table 2, and Figure 1. Clear and consistent differences in susceptibility were observed across both the test agents and the pathogen species.

**FIGURE 1:**
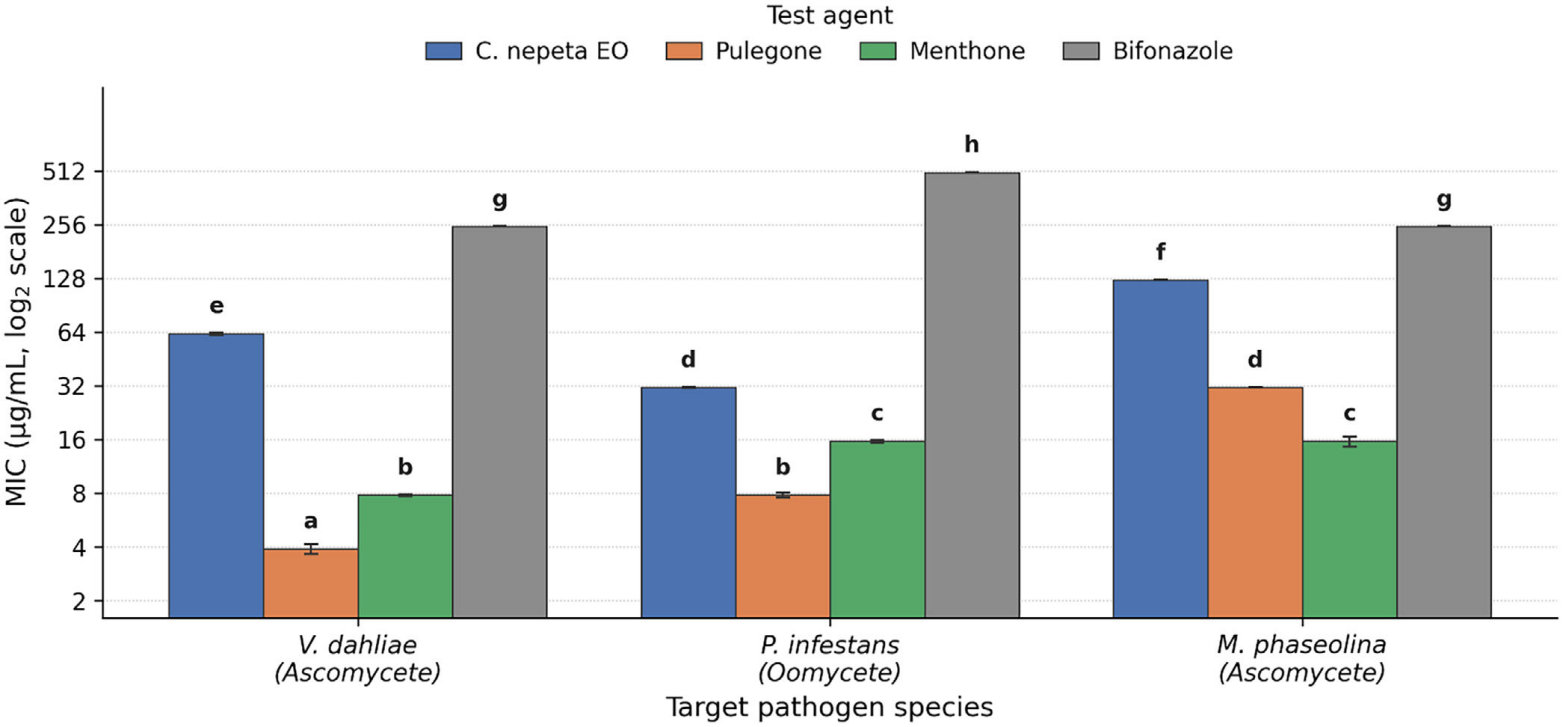
Minimum inhibitory concentrations (µg/mL, log2 scale) of *C. nepeta* essential oil, pulegone, menthone and the reference antifungal bifonazole against *V. dahliae*, *P. infestans*, and *M. phaseolina*. Each pathogen forms a cluster of four bars, one per test agent, color‐coded consistently across clusters. Bars are means of *n* = 3 independent experiments and error bars show ± SD; a lower MIC indicates greater potency. Bars not sharing a letter differ significantly (one‐way ANOVA followed by Tukey's HSD, *p* < 0.05). Carvacrol and thymol are discussed in the text as literature benchmarks but were not assayed in this study and are therefore not plotted. *P. infestans* is an oomycete (Stramenopiles) and not a true fungus, so its values represent anti‐oomycete rather than antifungal activity.

The whole EO inhibited growth of all three pathogens, with *P. infestans* emerging as the most sensitive organism (MIC = 31.25 µg/mL), followed by *V. dahliae* (MIC = 62.5 µg/mL) and *M. phaseolina* (MIC = 125 µg/mL). The fourfold range across pathogens indicates that EO susceptibility is strongly species‐dependent, reflecting differences in cell wall architecture, membrane composition, and passive exclusion mechanisms.

Strikingly, both isolated major constituents substantially outperformed the whole EO against all three pathogens. Pulegone was the most potent agent overall: against *V. dahliae*, it achieved an MIC of 3.90 µg/mL; against *P. infestans*, an MIC of 7.81 µg/mL. Menthone exhibited MIC values of 7.81 µg/mL (*V. dahliae*) and 15.62 µg/mL (*P. infestans*). Against *M. phaseolina*, the pattern was reversed: menthone (MIC = 15.62 µg/mL) outperformed pulegone (MIC = 31.25 µg/mL); both isolated constituents exhibited markedly lower MIC values than the whole EO (MIC = 125 µg/mL) against this pathogen. The antifungal standard bifonazole showed weak inhibitory activity against all three pathogens tested, with MIC values of 250 µg/mL against *V. dahliae*, 500 µg/mL against *P. infestans*, and 250 µg/mL against *M. phaseolina*.

The observed MIC values place *C. nepeta* EO and its major constituents among the most active monoterpene‐rich EOs reported against these phytopathogens. Particularly noteworthy is the activity of pulegone against *V. dahliae*, which approaches potency levels commonly associated with synthetic fungicidal compounds and highlights the potential of this molecule as a lead structure for future biopesticide development.

Carvacrol and thymol, the most extensively studied antifungal EO compounds, provide essential benchmarks. From the literature, carvacrol and thymol both exhibit strong anti‐oomycete activity against *P. infestans*: Deweer et al. reported that savory and oregano EOs (both carvacrol‐rich) achieved anti‐oomycete activities approaching that of copper sulfate, while thyme EO (thymol‐rich) showed comparable activity [44]. Structurally, carvacrol and thymol are phenolic monoterpenes whose hydroxyl groups confer both proton‐donating capacity (protonophore activity) and hydrogen‐bonding interactions with membrane phospholipids, mechanisms distinct from, but potentially overlapping with, the ketone‐based membrane disruption and Michael acceptor reactivity of pulegone and menthone [45, 46].

For *V. dahliae*, published MIC values for carvacrol and thymol in comparable broth microdilution assays span a range of approximately 30–250 µg/mL. The pulegone MIC of 3.90 µg/mL observed here would represent a substantial improvement over this range if confirmed by replicate assays, positioning the pulegone/menthone chemotype of *C. nepeta* as among the most potent non‐phenolic antifungal EO fractions reported for this pathogen. This comparison is most meaningful when assay conditions (medium, inoculum, and readout) are aligned, which is another argument for standardized reporting in EO antifungal research.

### 3.3 Differential Pathogen Susceptibility

The activity of oxygenated monoterpenes against fungi and oomycetes arises from several overlapping mechanisms rather than from a single molecular target, a feature that distinguishes them from site‐specific synthetic fungicides and limits the likelihood of resistance selection. Lipophilic monoterpenes partition into the plasma membrane, increase bilayer fluidity and dissipate the electrochemical proton gradient, causing leakage of cytoplasmic solutes; in true fungi they additionally lower ergosterol content and impair the function of membrane‐anchored enzymes, and the resulting loss of membrane potential secondarily promotes mitochondrial dysfunction and oxidative stress [44, 47, 48, 49].

The greatest sensitivity was recorded for *P. infestans*, both to the whole oil (MIC = 31.25 µg/mL) and to the two reference constituents. This organism is an oomycete, phylogenetically closer to brown algae than to true fungi: Its cell wall is built from cellulose and β‐1,3/β‐1,6‐glucans rather than chitin, and its plasma membrane contains fucosterol rather than ergosterol. The mechanisms most often invoked to explain the antifungal activity of monoterpenes, namely ergosterol depletion and disruption of ergosterol‐dependent raft domains, therefore cannot account for the activity observed here. A sterol‐independent perturbation of phospholipid organization is the more plausible explanation, particularly if oomycete membranes lack the sterol‐mediated regulatory capacity that allows true fungi to compensate for increased fluidity when challenged with lipophilic agents [50, 51]. This interpretation is consistent with the broad susceptibility of *P. infestans* to chemically diverse essential‐oil constituents, both phenolic and non‐phenolic, reported by Deweer et al., which is difficult to reconcile with a sterol‐specific mode of action [44].

Conversely, *M. phaseolina* was consistently the least susceptible organism (EO, 125 µg/mL; pulegone, 31.25 µg/mL). This species produces abundant microsclerotia in which tightly packed hyphal cells are enclosed within a thick matrix heavily melanized with 1,8‐dihydroxynaphthalene (DHN) melanin, a hydrophobic polymer that forms a physical barrier and adsorbs lipophilic compounds, reducing the concentration that reaches the plasma membrane [52, 53]. Comparable melanin‐mediated protection has been described in other melanized phytopathogens exposed to essential‐oil constituents.

The reversal of relative potency observed within this pathogen, menthone (15.62 µg/mL) being more active than pulegone (31.25 µg/mL), is structurally informative. The Michael reactivity that favors pulegone against the other two organisms may be a liability here: *M. phaseolina* maintains high intracellular concentrations of glutathione and related thiols as part of its constitutive oxidative‐stress response, conditions under which an electrophilic enone is readily conjugated and inactivated [54, 55]. The chemically inert menthone would then sustain a higher effective intracellular concentration. This remains a hypothesis; glutathione‐depletion experiments would provide a direct test.

### 3.4 Structural Basis of the Difference Between Pulegone and Menthone

The differential potency of pulegone and menthone across the three test pathogens indicates a constitutional structure‐activity relationship that the published *C. nepeta* literature has not previously addressed. The relationship is constitutional: pulegone and menthone differ in connectivity, through the presence or absence of the C4═C8 double bond, and the argument developed below turns on that difference alone.

Pulegone (p‐menth‐4(8)‐en‐3‐one) contains an α,β‐unsaturated cyclic ketone (enone) in which the exocyclic C4═C8 isopropylidene double bond is conjugated with the C3 carbonyl (Figure 2A). This enone constitutes a Michael acceptor: an electrophilic site susceptible to nucleophilic 1,4‐addition. Through this mechanism, pulegone can form covalent adducts with biological nucleophiles including cysteine and lysine residues in enzyme active sites, glutathione (GSH), and other thiol‐containing metabolites. This Michael reactivity also underlies pulegone's well‐characterized hepatotoxicity and may explain its superior potency against organisms with limited thiol‐quenching capacity [30, 41].

**FIGURE 2:**
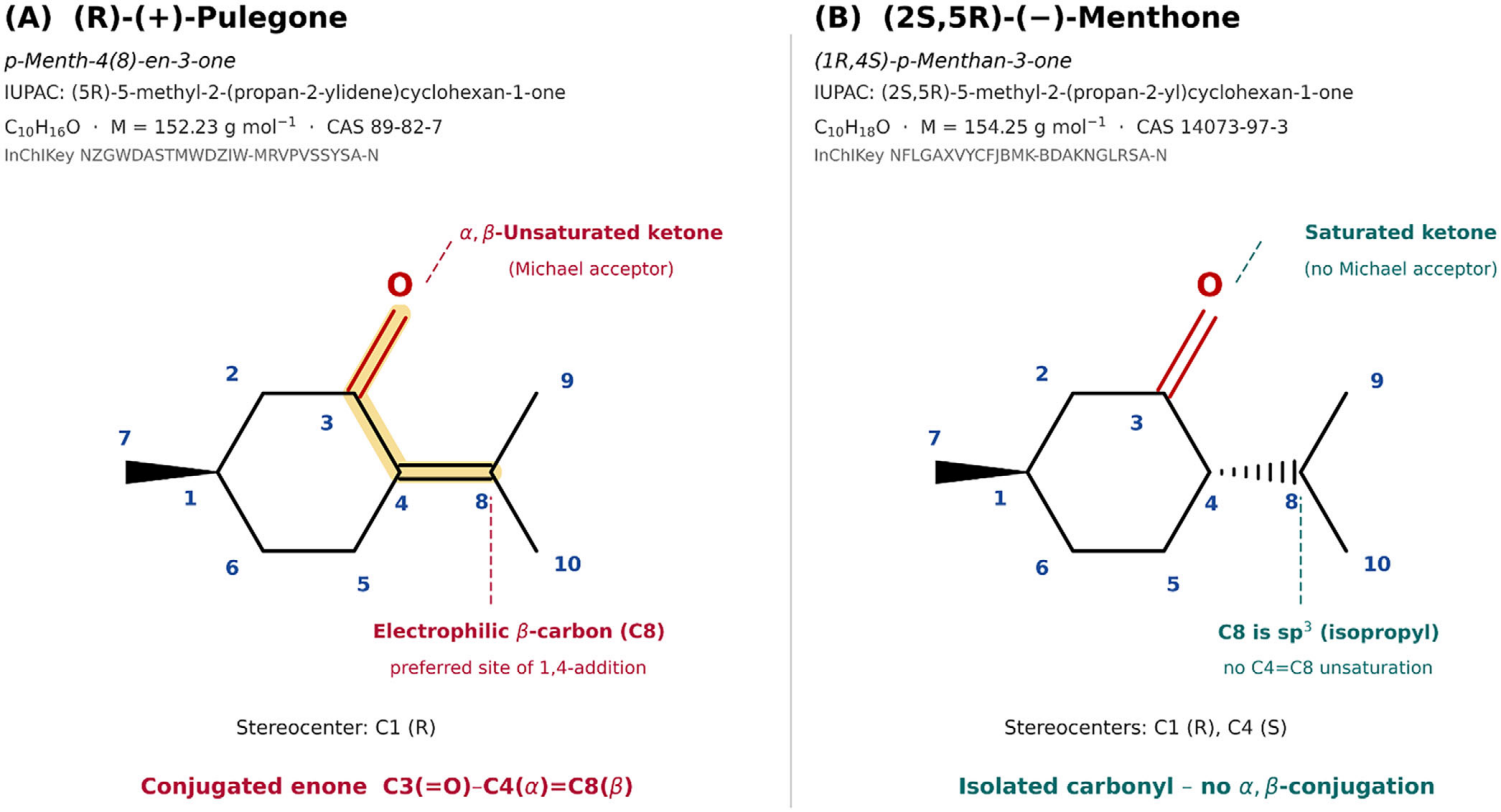
Structures of the two ketones assayed as isolated constituents, redrawn to correct geometry. In both panels the C1 methyl group and the C4 substituent lie on the same (para) axis of the p‐menthane ring, and every bond angle at an sp^2^ center (C3 and C4 in A, C3 in B, and the exocyclic C8 in A) is 120°; the ring is drawn as a regular hexagon with all C─C─C angles of 120°. (A) (R)‐(+)‐Pulegone (C10H16O; M = 152.23 g mol^−^^1^; CAS 89‐82‐7; InChIKey NZGWDASTMWDZIW‐MRVPVSSYSA‐N), p‐menth‐4(8)‐en‐3‐one, IUPAC name (5R)‐5‐methyl‐2‐(propan‐2‐ylidene)cyclohexan‐1‐one, carries a conjugated enone C3(═O)─C4═C8 (shaded), conferring Michael‐acceptor character with C8 as the electrophilic β‐carbon. (B) (2S,5R)‐(−)‐Menthone (C10H18O; M = 154.25 g mol^−^^1^; CAS 14073‐97‐3; InChIKey NFLGAXVYCFJBMK‐BDAKNGLRSA‐N), (1R,4S)‐p‐menthan‐3‐one, IUPAC name (2S,5R)‐5‐methyl‐2‐(propan‐2‐yl)cyclohexan‐1‐one, differs solely by saturation of C4─C8, leaving the carbonyl unconjugated. Both structures have been redrawn: each comprises exactly ten carbon atoms (C1─C10) and one oxygen atom, in agreement with the molecular formulae, and both were verified computationally against the InChIKeys given above. Locants on the drawings follow p‐menthane numbering, in which C1 bears the methyl group and C4 the isopropylidene or isopropyl group; locants in the IUPAC substitutive names follow cyclohexanone numbering, in which the carbonyl carbon is C1. Bold wedge = above the ring plane; hashed wedge = below. The stereodescriptors apply to the commercial reference compounds used in the bioassays only; the configuration of the corresponding essential‐oil constituents was not determined (Section 2.2).

Menthone (p‐menthan‐3‐one) is the corresponding saturated analogue, in which the C4═C8 double bond is absent (Figure 2B). The isolated ketone carbonyl of menthone is a far weaker electrophile, making menthone essentially nonreactive as a Michael acceptor. Its antifungal activity therefore relies primarily on physical membrane partitioning, disruption of membrane fluidity through hydrophobic insertion, and non‐covalent inhibition of membrane‐associated enzyme complexes. Its superior activity against *M. phaseolina* relative to pulegone, precisely the organism with the most robust reactive‐thiol defense, is consistent with this interpretation: menthone's non‐covalent mechanism is not compromised by the high GSH environment that appears to limit pulegone's effectiveness against this pathogen [56, 57].

It should be emphasized that the comparison drawn here is constitutional and not configurational. Both reference compounds were assayed as single, supplier‐certified stereoisomers, so the comparison is internally consistent; but the present data cannot establish whether the antipodal stereoisomers would behave differently, and no enantioselective structure–activity relationship is claimed. Enantiomer‐resolved potency data would be required for that purpose and are proposed as the immediate continuation of this work.

### 3.5 Whole Essential Oil Versus Individual Reference Constituents

The finding that pulegone and menthone individually outperform the whole EO by 2‐ to 16‐fold against all three pathogens is the principal result of this study. Three nonexclusive explanations may be considered.

- *Antagonistic interactions within the matrix*. Compounds sharing membrane targets, or acting through partially overlapping modes of action, may compete for binding sites or interfere with one another's access to them. The minor constituents of this oil, neoisomenthol (9.25%), limonene (3.30%) and α‐ and β‐pinene (0.38% each), have limited intrinsic antifungal activity at these concentrations, but may alter the biophysical properties of the membrane in ways that reduce the target engagement of the two dominant ketones. Such compositional antagonism is frequently invoked in the essential‐oil literature but rarely quantified; fractional inhibitory concentration (FIC) index determination is required to characterize it formally [58, 59].
- *Differences in dispersibility*. The whole oil is a lipophilic mixture whose aqueous dispersibility is governed by its overall hydrophobicity. Its monoterpene hydrocarbon fraction (4.36%) raises the global LogP and may reduce effective availability in aqueous growth medium relative to individual compounds dissolved in a polar solvent at the same nominal concentration, systematically underestimating whole‐oil potency in broth microdilution assays.
- *Quenching of the electrophilic site*. Nucleophilic minor constituents could partially consume the Michael acceptor of pulegone within the matrix before it reaches its biological target, which would constitute an in vitro artifact rather than genuine biological antagonism.

Distinguishing between these possibilities requires FIC index analysis combined with fractionation and gas‐chromatographic quantification of residual pulegone. Neither was performed here, and the term antagonism is therefore used descriptively, to denote the observed departure from additivity, rather than as a formally demonstrated interaction.

## 4 Conclusions

*Clinopodium nepeta* EO from northeastern Morocco belongs to the pulegone/menthone chemotype, with these two oxygenated monoterpene ketones accounting for approximately 83.6% of the oil composition. The oil and its isolated major constituents inhibit growth of three economically important plant pathogens, *V. dahliae*, *P. infestans* (oomycete), and *M. phaseolina*, in vitro, with MIC values as low as 3.90 µg/mL (pulegone vs. *V. dahliae*). Across all three pathogens, the isolated ketone constituents outperformed the whole oil by approximately 2‐ to 16‐fold, indicating antagonistic interactions within the complex essential‐oil matrix and suggesting that constituent‐enriched formulations could substantially exceed raw‐oil potency in field applications. The differential selectivity of pulegone and menthone, with pulegone dominant against the true fungi and menthone superior against *M. phaseolina*, is consistent with their structural distinction as an α,β‐unsaturated electrophilic ketone versus a saturated, essentially non‐Michael‐active ketone, and with plausible pathogen‐specific differences in thiol‐mediated detoxification capacity, melanin‐based exclusion mechanisms, and membrane sterol composition (including the oomycete‐specific sterol system of *P. infestans*). Beyond their immediate phytopathological relevance, these findings contribute to a broader understanding of how individual essential‐oil constituents interact with microbial targets and demonstrate that the biological activity of a complex natural mixture cannot always be predicted from its overall composition. This mechanistic insight provides an important conceptual basis for the future development of evidence‐based botanical pesticides. These findings support the identification of pulegone and menthone as priority candidates for botanical biopesticide development, with a clear pathway toward formulation optimization, in planta efficacy validation, and regulatory risk assessment, steps that are indispensable before any responsible recommendation for field deployment. That assessment must in particular address the documented hepatotoxicity of pulegone, whose CYP450‐mediated bioactivation to menthofuran underlies the restrictions placed on its use in food flavorings and cosmetics in the European Union and in several other jurisdictions [60, 61]. The most tractable near‐term application is therefore soil treatment directed against the soilborne pathogens *V. dahliae* and *M. phaseolina*, in non‐edible crops or by pre‐season incorporation, where residues on consumed plant parts are not at issue. Beyond their immediate agricultural relevance, the contrasting bioactivity profiles of pulegone and menthone provide a molecular blueprint for probing the fundamental chemistry–biology interface in plant–pathogen interactions, with potential implications for the discovery of next‐generation resistance‐resilient crop protection agents. Two limitations bound these conclusions. First, the chromatographic analysis was performed on an achiral phase, so the constituents of the oil are characterized at the level of constitution only; the structure–activity relationship advanced here rests on the presence or absence of the α,β‐unsaturation and not on absolute configuration, and enantioselective GC coupled with bioassay of the individual antipodes will be required before any configurational structure–activity claim can be entertained. Second, two minor peaks together representing 1.08% of the oil could not be assigned with confidence and are reported as unidentified.

## Author Contributions

**Mohamed Taibi**: conceptualization, methodology, investigation, formal analysis, writing – original draft. **Amine Elbouzidi**: conceptualization, methodology, formal analysis, writing – original draft. **Mounir Haddou**: investigation, data curation, writing – review and editing. **Abdessamad Beraich**: investigation (GC–MS analysis). **Abdellah Baraich**: investigation (antimicrobial assays). **Reda Bellaouchi**: methodology, validation. **Mohamed Addi**: resources, botanical authentication of the plant material. **Khalid Chaabane**: supervision, validation. **Bassem Jaouadi**: conceptualization, supervision, project administration, funding acquisition, writing – review and editing. **Nadia Zarai**: investigation, validation. **Bouchra El Guerrouj**: supervision, resources. **Abdeslam Asehraou**: supervision, project administration, writing – review and editing. All authors have read and approved the final version of the manuscript.

## Funding

This work was supported by the EU‐PRIMA Section 2—Multi‐topic 2023, Project NOVISHPAK, (the PRIMA programme is supported under Horizon 2020, the European Union's Framework Programme for Research and Innovation). Additional funding was provided by the Multilateral Project Partenariats Hubert Curien (PHC)‐Maghreb 2025 Program (NGS‐ENZin 2025‐2027, Campus France Code: 51730VM & PHC Code: 25MAG38). This program is a collaboration between French institutions—the Ministry for Europe and Foreign Affairs (MEAE), the Ministry of Higher Education, Research and Innovation (MESRI), and the National Centre for Scientific Research (CNRS)—the Tunisian Ministry of Higher Education and Scientific Research (MESRS) through its Directorate General for Scientific Research (DGRS), and the Moroccan Ministry of Higher Education, Scientific Research and Innovation (MESRI) through its National Center for Scientific and Technical Research (CNRST). The funders had no role in study design, data collection and analysis, decision to publish, or preparation of the manuscript.

## Conflicts of Interest

The authors declare no conflicts of interest.

## Artificial Intelligence Usage Statement

An AI‐based language tool was used for English language editing.

## Supporting information




**Supporting File 1**: cbdv71781‐sup‐0001‐SuppMat.docx.

## Data Availability

The data supporting the findings of this study are available within the article and its Supporting Information. The total‐ion GC–MS chromatogram is provided as Figure S1 and the identified constituents are listed in Table 1.
