# Supplementary material for: Clinopodium nepeta Essential Oil and Its Major Constituents Pulegone and Menthone: Composition and Activity Against Verticillium dahliae, Phytophthora infestans, and Macrophomina phaseolina
Source: Chem Biodivers. 2026 Sep 30;23(10):e71781. doi: 10.1002/cbdv.71781 (PMC13626859; doi:10.1002/cbdv.71781)
Supplement: Supplementary file 1 [file CBDV-23-e71781-s001.docx]

**Figure S1.** GC–MS chromatogram of *C. nepeta* essential oil obtained by hydrodistillation. X-axis: retention time (min); Y-axis: total ion current abundance. All 14 resolved peaks are numbered above the trace, following the numbering of Table 1; 12 of them were identified and together account for 98.92% of the total composition, while peaks 9 and 11 are reported as unidentified. Peaks for pulegone (42.16%, peak 12) and menthone (41.43%, peak 8) are the two dominant signals of the trace.
